# Behavioral predictors of autism recurrence are genetically independent and influence social reciprocity: evidence that polygenic ASD risk is mediated by separable elements of developmental liability

**DOI:** 10.1038/s41398-019-0545-z

**Published:** 2019-08-22

**Authors:** Alexa Pohl, Warren R. Jones, Natasha Marrus, Yi Zhang, Ami Klin, John N. Constantino

**Affiliations:** 1 Washington University, St. Louis, Missouri USA; 2 Marcus Autism Center, Emory University and Children’s Healthcare of Atlanta, Atlanta, GA 30322 USA; 3 Department of Psychiatry, Washington University in St. Louis, St. Louis, MO USA; 4 Departments of Psychiatry and Pediatrics, Washington University in St. Louis, St. Louis, MO USA

**Keywords:** Genetics, Neuroscience

## Abstract

The preponderance of causal influence on total population attributable risk for autism is polygenic in nature, but it is not known how such liability engenders the development of the syndrome. In 348 epidemiologically ascertained toddler twins, we explored associations between autistic traits and three robust, highly heritable predictors of familial autism recurrence: variation in attention, motor coordination, and parental autistic trait burden. We observed that these predictors—despite collectively accounting for over one third of variance in clinical recurrence—are genetically independent in early childhood, and jointly account for a comparable share of inherited influence on early reciprocal social behavior in the general population. Thus, combinations of what are otherwise discrete, inherited behavioral liabilities—some not specific to autism—appear to jointly mediate common genetic risk for autism. Linking genetic variants and neural signatures to these independent traits prior to the onset of the development of autism will enhance understanding of mechanisms of causation in familial autistic syndromes. Moreover, ongoing biomarker discovery efforts will benefit from controlling for the effects of these common liabilities, which aggregate in individuals with autism but are also continuously distributed in “controls”. Finally, early inherited liabilities that participate in the early ontogeny of autistic syndromes represent parsimonious intervention targets for polygenic forms of the condition, and represent candidate trans-diagnostic endophenotypes of potential relevance to a diversity of neuropsychiatric syndromes.

## Introduction

Although molecular genetic studies exploring the impact of highly deleterious de novo mutations have provided deep insights into pathogenesis for rare forms of autism spectrum disorder (ASD), a vast share of the population-attributable-risk for autism (upwards of 80% of the total causal variance) is traceable to inheritance^1,2^, which by definition, is not encompassed by the effects of de novo mutations. Understanding the mechanisms through which inherited susceptibilities—which are typically polygenic and additive—give rise to autism may be enhanced by tracing their respective influences to parsimonious endophenotypes that are influenced by specific components of polygenic liability. Although robust prior attempts to trace a number of putative neuropsychiatric endophenotypes (including psychophysiologic measurements presumed “closer” to discrete biological processes than overt behavior) to molecular genetic underpinnings have been sobering in terms of effect size of association, a recent convergence of findings from family studies of autism spectrum disorder (ASD) has identified a small number of heritable, school-aged behavioral correlates of autism recurrence^2^ that collectively account for a substantial share of variation-in-outcome of close relatives of autism probands. Specifically, we and others have reported that impairment in motor coordination^3–5^, attention^6–8^; and parental autistic trait burden^9,10^ predict autism, jointly accounting for one third to one half of its recurrence in families. Remarkably, the findings related to hyperactivity and motor coordination impairment corroborate a number of reported associations between cerebellar dysfunction and autism^11–13^. Although behavioral predictors of autism recurrence (BPARs) co-aggregate by definition in individuals with familial autistic syndromes, it is not yet established whether they are causally linked to one another in the general population, especially early in life, during the developmental period when the behavioral signs and symptoms of autism first manifest. Demonstration of their genetic independence during this phase of development would inform an emerging model of causation in autism, namely that familial forms of the condition arise from specific combinations of early, species-typical variations in behavior, each independently inherited, and none of which individually give rise to the syndrome^2^.

Here, we examined associations between three BPARs and both their individual-associaations and joint-associations with deficits in reciprocal social behavior—a defining feature of the autistic syndrome—in early childhood. This study capitalized upon the availability of two epidemiologic twin cohorts (from Missouri and California) in which (a) parental autistic trait burden had been ascertained as an index of background genetic liability for autistic social impairment in their offspring^10,14^, and (b) in which the offspring were also assessed for measures of attentional problems, motor coordination, and social developmental outcome, specifically within the age range from 36 to 48 months. The twins were enrollees in a prospective study beginning at 18 months of age (see below). Autistic trait variation in parents and reciprocal social behavior in their children were ascertained using a measure of autism-related variation in human social development which quantifies aspects of social reciprocity (i) whose deficiency characterizes autism^15,16^, (ii) which are highly stable over the life course^17^, and (iii) which aggregate in unaffected family members of probands with autism^10,18^. Genetic epidemiologic research has demonstrated that general population variation in the characterizing features of autism—principally variation in reciprocal social behavior—exhibits marked causal overlap with additive genetic liability for clinical autistic syndromes^18,19^.

## Materials/subjects and methods

### Participants

The twin sample was epidemiologically ascertained through the Missouri Family Register (MFR), a birth records registry maintained by the WUSM Department of Psychiatry in collaboration with the State of Missouri as described in detail in Constantino et al.^20^, and simultaneously through the California Twin Register. The consenting family member was required to be the legal guardian and primary caregiver and to speak fluent English. This study protocol was approved by the Washington University School of Medicine (WUSM) Human Research Protection Office (IRB), HRPO #201208010, as well as by Stanford University’s Institutional Review Board, the State of California’s Committee for the Protection of Human Subjects’, and the Missouri Division of Vital Records. The parents of all participants gave informed consent prior to each assessment.

Based upon Missouri Family Register data, we were able to contact 330 eligible families of Missouri twins in the specified age range during the calendar years 2011–2013. Of these, 180 enrolled in the Early Reciprocal Social Behavior study (complete details described in ref. ^20^), with complete or partially complete data sets acquired from 160 pairs at 18 months, 143 pairs at 24 months, 131 pairs at 36 months and 112 pairs at 48 months. Of the original 160, four pairs were removed for quality concerns. Following selection of one twin per pair from the remaining 156 (for inclusion in regression and correlation analyses), 89 had complete data for all BPARs ascertained in the study. There were no statistically significant differences in mean scores for early variation in reciprocal social behavior between the children with complete data and those with missing or incomplete data.

**Fig. 1:**
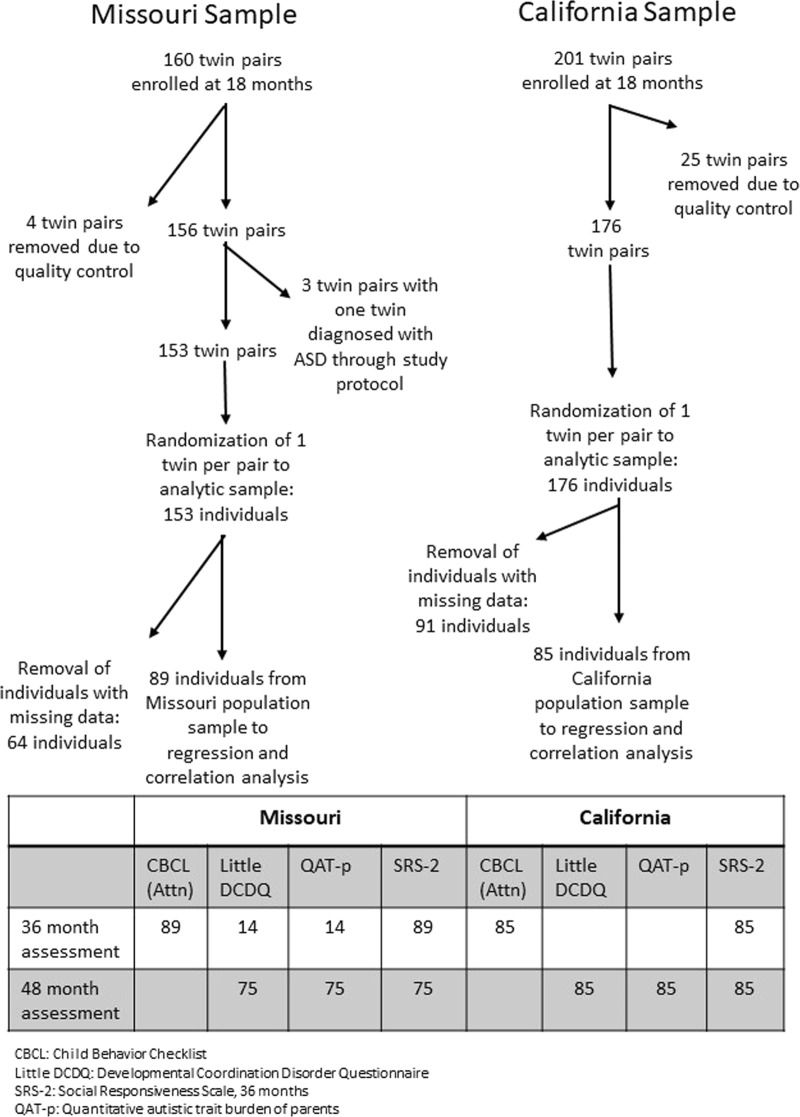
Study flow diagram and table of measurements as a function of longitudinal follow-up

For the California site, all Hispanic families with living twins born between January 1, 2012 and December 31, 2012 who resided in California were identified from California birth records (*n* = 2873). Of these, 455 responded to initial contact (i.e., requested a screening interview) and were targeted for participation. Of these, 313 families enrolled, 81 families opted out, and a total of 61 families were ineligible (not fluent in English *n* = 52; moved out of state *n* = 3; not of Hispanic Ancestry *n* = 3; died *n* = 2; and parents did not have legal guardianship of twins *n* = 1). Of the 313 enrolled families, 201 completed data collection at 18 and 24 months; among these, 25 pairs were removed due to issues with data quality. Following selection of one twin per pair (of the remaining 176) at random for inclusion in regression and correlation analyses, slightly over half were missing one or more of the measures, leaving 85 with complete data for all BPARs ascertained in the study. Figure 1 depicts the derivation of the current sample and the measures that were administered at each study time point.

The resulting accumulated sample of twins was deemed more than adequate to test the study’s principal hypotheses, i.e., to detect heritability on the order of 0.30 or higher, to detect bivariate correlations between early behavioral traits on the order of 0.15 or higher, and to detect joint effects of early behavioral predictors on general population early childhood reciprocal social behavior at a level of 15 per cent of the total variance, which is under half of the total variance explained by these traits in clinical recurrence studies, as inferred from the previously published studies cited in the Introduction. To examine the sample for any possible selection bias, families who were included in the sample were compared to those who were not included in the sample with information from birth records. Analyses indicated that families included in the sample in both states had higher socioeconomic status (as measured by higher parental education attainment and lower proportion of mothers who used government programs), with participation by older parents. In the California sample, families were more acculturated (as measured by proportion of mothers who were born in the U.S.) and had a higher proportion of multiracial fathers in comparison to families who did not participate. There were no other appreciable differences between families who participated and those identified in the respective epidemiologic sampling frames (details for the Missouri sample are provided in Constantino et al.^20^; for the California sample all corresponding information is presented in Supplementary Table 1).

### Zygosity confirmation

Zygosity was determined by the Goldsmith Child Zygosity Questionnaire, which corresponds to DNA marker/blood type determinations of zygosity in 94.8% of cases. The questionnaire was administered during a phone interview with the twins’ biological mother or father. Correspondence between the questionnaire-based zygosity determination and genotypic assignment, using DNA acquired by buccal swab, was tested for a randomly selected subset of families (*n* = 30 twin pairs) and in all cases positively confirmed the questionnaire results^20^.

### Measures

#### Social responsiveness scale 2nd Ed (SRS-2)

The SRS-2 is a 65-item quantitative measure of autism-related variation in reciprocal social behavior. Scores on the SRS are highly heritable, continuously distributed in the general population and exhibit trait-like stability over the course of life from preschool through adulthood^17^. Ratings on the SRS-2 for individuals with autism spectrum disorder are, on average, three standard deviations higher than those for unaffected individuals in the general population^15,16^. The 2nd edition includes a preschool form for ages 2.5–4 years, a 4–18-year-old version, and an adult form for ages 19 and older. Toddler twins had the SRS-2 completed by a parent (usually the mother) at 3 years and again at 4 years of age, while parents rated one another using the adult form (i.e., by spouse-report). Adult ratings on the SRS exhibit as strong a level of heritability as are observed for the ratings of children^21^.

#### Child behavior checklist (CBCL)

The Achenbach Scales of Empirically Based Assessment comprise an extensively validated parent-report and/or teacher-report rating system for behavior problems during preschool and childhood. The CBCL preschool forms (ages 1.5–5 years) yield seven syndrome scores; in this study the Attention Problems scale was used for quantitative ascertainment of traits and symptoms characteristic of Attention Deficit Hyperactivity Disorder (ADHD), as was implemented for school-aged children in an autism recurrence study reported by Mous et al.^6^.

#### Little developmental coordination disorder questionnaire (Little DCDQ)

The Little Developmental Coordination Questionnaire is a parent report measure that screens for motor coordination difficulties in 3-year-old and 4-year-old children. It consists of 15 items, and ascertains both fine and gross motor skills. The Little DCDQ measures functional skills in several contextual areas across home and preschool environments and during play activities^22^.

### Data analysis

**Fig. 2:**
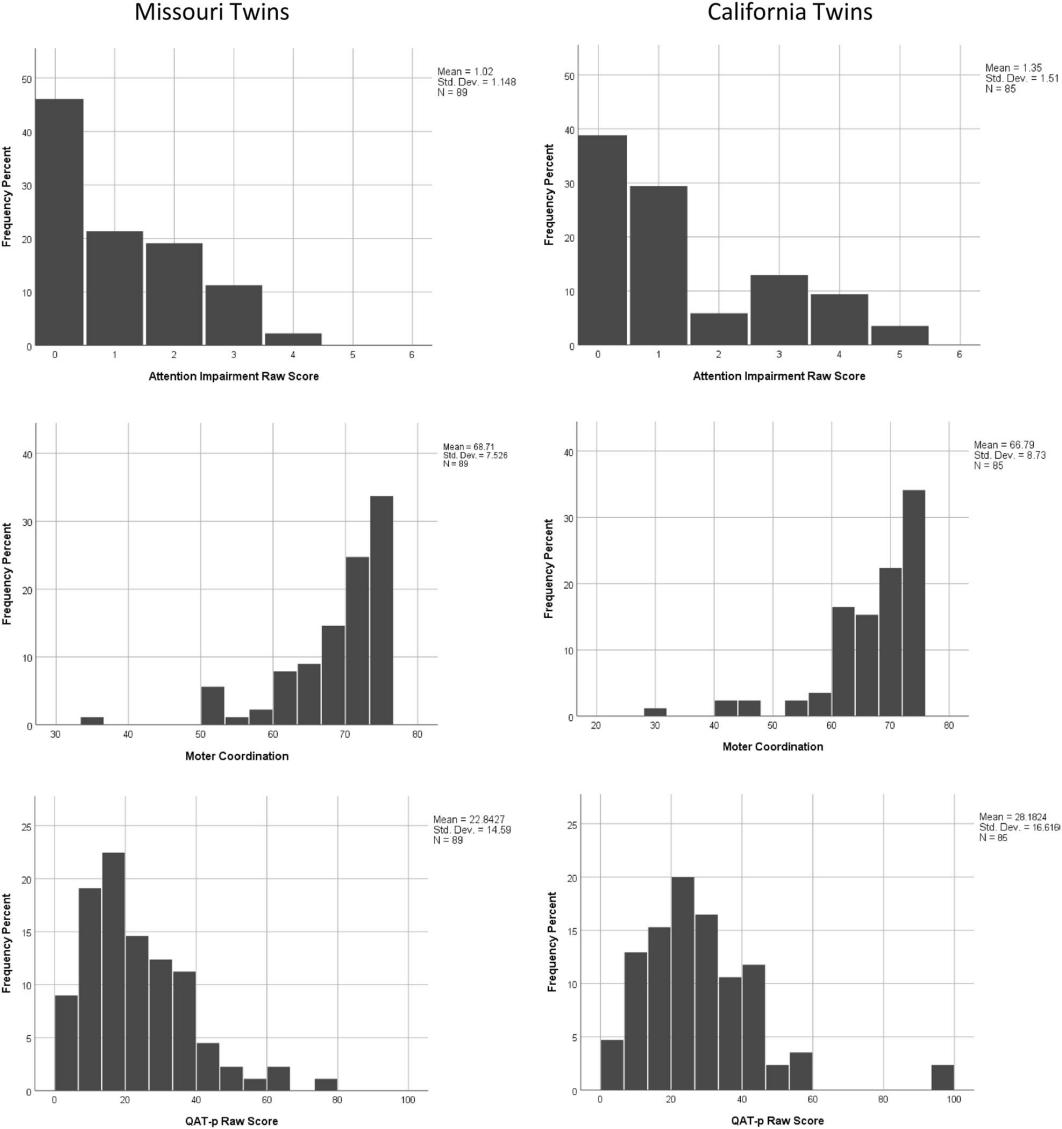
Histograms depicting trait distributions of behavioral predictors of autism recurrence examined in this study

Histograms depicting the distributions of scores for child-specific ratings implemented among the twins are provided in Fig. 2, and established the suitability of application of standard parametric analytic methods to the data. Quantitative autistic trait burden of parents (QAT-p) was estimated as the average total Social Responsiveness Scale score of each child’s parents. We examined heritability of the recurrence predictors by applying Falconer’s method to the respective twin-twin correlations of monozygotic and dizygotic twins; this analysis excluded opposite sex twin pairs and pairs with uncertain zygosity; the estimates were derived from 66 MZ pairs and 58 DZ pairs with complete data for all ascertained BPARs). Next, we calculated bivariate correlations between the measures, and estimated their individual and joint association with variation in reciprocal social behavior at 36 and 48 months of age using linear regression analysis.

## Results

In this sample we observed strong trait heritability for the measure of autism-related variation in reciprocal social behavior implemented in this study (i.e. the primarydependent/longitudinal outcome variable), as described in prior research; .91 for both males and females. The heritabilities of child-specific behavioral *predictors* of autism recurrence (BPARs) measured in the twins were as follows: ADHD traits: 0.53 for males, 0.58 for females; developmental motor coordination disorder traits: 0.41 for males, 0.55 for females. As shown in Table 1, the monozygotic twin correlations were extremely high for developmental motor coordination disorder traits and less pronounced for ADHD traits. Females manifested a slightly higher influence of non-shared environmental influences on SRS-2 scores than their male counterparts.

**Table 1:** Falconer’s heritability and twin-twin correlations for two behavioral predictors of ASD recurrence (Attention Problems and variation in Motor Coordination) and autism-related variation in reciprocal social behavior in the fourth year of life

	Attention problem (CBCL)	Motor coordination (Little DCDQ)	Reciprocal social behavior (SRS-2)
MZM twin-twin correlation	.527	.937	.784
DZM twin-twin correlation	−.013	.734	.327
MZF twin-twin correlation	.583	.898	.913
DZF twin-twin correlation	−.081	.625	.352
Heritability (males)	.53*	.41	.91
Heritability (females)	.58*	.55	.91*

The analysis excluded opposite sex twin pairs and pairs with uncertain zygosity; they are derived from 66 MZ pairs (34 male-male and 32 female-female) and 58 DZ pairs (30 male-male, 28 female-female) with complete data for these three variables

*For heritability estimate >1, MZ concordance rate is used by convention

*MZM* monozygotic male

*DZM* dizygotic male

*MZF* monozygotic female

*DZF* dizygotic female

*CBCL* child behavior checklist

*Little DCDQ* developmental coordination disorder questionnaire

*SRS-2* social responsiveness scale, 36 months

Cross-trait associations are summarized in Table 2: in this epidemiologic sample, none of the measured BPARs accounted for more than 5 per cent of the variation in any other BPAR. Thus to the extent that each is influenced by additive genetic factors, the extent to which their inherited variation overlapped was negligible in this sample. Bivariate cross-twin cross-trait correlations encompassing attentional impairment, QAT-p, and motor coordination (the recurrence predictors) were also uniformly non-statistically significant. Each of the BPARs, however, was moderately and highly statistically significantly predictive of variation in reciprocal social behavior as measured by the Social Responsiveness Scale at 36 and 48 months. Although neither of the child-specific BPARs exhibited statistically significant associations with the measurements of quantitative autistic traits of their parents, they were predictive of the children’s own variation in quantitative autistic trait burden, the total of which was incrementally accounted for by (a) BPARs measured within the children (variation in attention and motor coordination) and (b) the quantitative autistic trait scores of their parents. In a linear regression analysis (Table 3), the three predictors jointly accounted for 35% of the variance in typical social developmental outcome, ascertained using the SRS-2. This proportion of variance in SRS-2 measurements was highly statistically significant, and its magnitude was in keeping with that observed for within-family recurrence of *clinical* autistic syndromes^6^ when predicted by these same factors.

**Table 2:** Matrix depicting within-individual correlations (Pearson’s *r*) between autism recurrence predictors in 174 general population twins (one twin selected at random from each pair)

	Attentional impairment	QAT-p	Motor coord	SRS 36 months	SRS 48 months
Attentional impairment	1				
QAT-p	.20	1			
Motor coordination	−.20	−.16	1		
SRS-2 (36 months)	.46	.38	−.36	1	
SRS-2 (48 months., *n* = 160)	.48	.28	−.35	.71	1

All correlations above 0.29 were statistically significant at *p* < .01; none of the bivariate associations between BPARs reached this threshold. Bivariate cross-twin cross-trait correlations encompassing attentional impairment, QAT-p, and motor coordination were uniformly non-statistically significant

*QAT-p* Quantitative Autistic Traits of Parents, as measured by the Social Responsiveness Scale, Adult Version

**Table 3:** Results of linear regression analysis examining the joint contribution of three behavioral predictors of autism recurrence (measured at 36-48 months) to variation in autism-related variation in early childhood reciprocal social behavior

Outcome modeled	Adj *R*^2^	BPAR	*B*	*t*	Sig	Δ Adj *R*^2^
SRS at 36 months	0.35	Biparental QAT	0.255	5.568	<0.001	0.06
		Variation in attentional impairment	0.355	3.996	<0.001	0.12
		Variation in motor coordination	−0.242	−3.830	<0.001	0.05
		Site	.079	1.252	0.212	0.00

Adjusted R square is reported for the full regression model, along with changes in adjusted R square that occur when a given individual behavioral trait is excluded from the model, and the result compared with that for the full model. A companion table for SRS outcome at 48 months (for which there were fewer twin pairs with complete data) yielded highly comparable results and is provided in Supplementary Table 2

*SRS* social responsiveness scale

## Discussion

In this study of 348 epidemiologically ascertained twins across two disparate U.S. populations, we observed that three established predictors of recurrence in autism, each of which are continuously distributed in the general population and moderately- to highly-heritable, jointly predicted autism-related variation in reciprocal social behavior. The three predictors, two ascertained in the children and one in their parents, were almost entirely uncorrelated with one another, each accounting for less than 5 per cent of the variance in the other two. *These data indicate that the total polygenic risk for autistic social impairment may be divisible into components that give rise to separate, earlier-contributing developmental liabilities, and if so this would have potentially far-reaching implications for understanding the manner in which genes, brain, and behavior are linked in autism*. Given that the largest share of the population-attributable-risk for autism is traceable to additive genetic influence^1,23,24^ clearer understanding of the manner in which inherited susceptibilities give rise to autism may require parsing their influence to intermediate phenotypes controlled by variation in specific, independent subsets of polygenic liability^2^. If autism arises from permutations and combinations of genetically independent, normally distributed precursor traits (each at or near the pathological end of their respective distributions in the population), it would radically modify understanding of the genetic structure of common autistic syndromes and would prompt a search for specific genetic and neurobiologic correlates of these and potentially other behavioral liabilities which index risk for autism when a) they fall at or near the pathological extreme of the normal distribution; and b) they are combined—within an individual—with one or more additional susceptibilities. Here, we observed substantial heritabilities for independent, child-specific BPARs in early childhood, with some degree of differential heritability for early attention and motor coordination as a function of sex. This latter observation is important to note because sexual dimorphisms in inherited behavioral liabilities underlying autism may contribute to sex-specific reduction in expression of genetic liability to ASD that has been observed among females in genetic epidemiologic research^18,25^ and that may account for the pronounced, universally observed sex ratio in autism.

Recently, using data involving a sub set of the Missouri twins, we demonstrated that yet another normally distributed behavioral trait under stringent genetic influence, the allocation of attention to socially-salient aspects of visual experience—as measured by tracking infants’ visual engagement with dynamic social scenes—constitutes an additional contributor to inherited liability to autism^20^. The precise relationship between low levels of eye-looking and face-looking (which predicts ASD recurrence among children at high familial risk) to the aspects of behavioral variation described in this communication is being actively explored in larger samples, but our prior study demonstrated that there exists a sub set of typically developing children in the general population who manifest low social visual engagement—at a level that would otherwise predict recurrence of autism in ASD-affected families—and who do *not* have social disability. The current data from epidemiologically ascertained twins across two U.S. regions add three additional independent predictors to what may be a finite set of early inherited liabilities which, singly, appear insufficient to engender autism, but when present at or near the pathological extreme of the respective general population distribution—and *in combination with* co-existing behavioral or genetic liabilities (including disruptive mutations)—may confer enough cumulative inherited susceptibility to engender autism when the total liability reaches or exceeds a threshold for clinical-level affectation.

There are clear implications of this model of additive genetic influence for the identification of autism biomarkers. If a common set of normally distributed traits mediates polygenic risk for autism, then failure to characterize these trait variations among controls in case-control studies will substantially confound the identification of genetic or neurobiologic biomarkers and could account for “missing heritability” in genotype-phenotype association studies of autism. When left unaccounted for, individual trait aggregations among control subjects may be almost as common as in autism, but of insufficient severity or *joint* aggregation to give rise to an autistic syndrome. This could also account for failures of polygenic risk correlates of common autism comorbidities (eg. ADHD) to index risk for autism, given that polygenic risk profiles are typically derived from case-control studies in which controls are not assessed for cross-trait symptom burden.

Although the ascertainment of quantitative autistic traits of parents reflects enduring aspects of inherited liability present from *before* the time of birth of the subjects, a limitation of the study is that the ascertainment of variation in ADHD and developmental motor coordination disorder symptomatology occurred at 36–48 months of age, which is somewhat later than the time when autistic syndromes first arise in most cases of ASD. The decision was made to defer the measurement of these traits to that time interval in this longitudinal study in order to optimize validity-of-assessment of these traits. There exist no established methods for earlier reliable quantitative characterization of these child-specific traits in the first and second year of life, before autistic syndromes are generally first appreciable, and this remains a high priority for future research, since their reliable assessment earlier in development would contribute to the identification of phenotypic indices of inherited liability that might be directly targeted by novel therapies to prevent or ameliorate the severity of ASD. An additional limitation is that in this prospective longitudinal study of epidemiologically ascertained 18 month old twins in two U.S. states, there were parameters of subject eligibility (requirement for English fluency of Hispanic parents) and levels of sample attrition that may have resulted in the exclusion of cases that would have qualified the estimates of association reported here. This possibility was mitigated by the broad, continuous distribution of variation in behavioral outcome represented by the children in the sample.

In conclusion, the observation that substantially heritable behavioral predictors of autism recurrence segregate independently in the general population—and make joint predictions for variation in typical social development of comparable magnitude to those for autism recurrence—supports the notion that familial autism is developmentally “fractionable,” but *not* according to symptoms that characterize the syndrome *afte*r it occurs, rather by discrete elements of genetically influenced liability that may be indexed by an array of *earlier*-appreciable neurobehavioral traits^26^. Some of these liabilities are not specific to ASD, and as such may relate to the autism “co-morbidities,” which may be important to construe as actual contributors to the cause and severity of autism itself. Linking genetic variants and neural signatures to these pleiotropic traits prior to the onset of the development of an autistic syndrome will enhance understanding of mechanisms of causation in familial autistic syndromes. Moreover, future research on the neurobiology of autism and related disorders will benefit from a focus on understanding why the *co-aggregation* of BPARs that are not specific to social abnormality (e.g., inattention and motor coordination) would jointly amplify risk for a syndrome of profound *social* disability, with timing of onset in the second year of life.

Finally, these data underscore the urgency of improving phenotypic characterization of these contributing traits in infancy. It has previously been shown that impairments in attention and reciprocal social behavior are substantially inter correlated later in development, i.e., among school-aged children both in the general population and among children with ASD^16,27^—in contrast to their dissociation in early childhood as observed here—suggesting that interactions between these and other early independent traits amplify cross-trait severity over the course of time and later childhood development^28,29^. Currently, although prodromal manifestations of both attention deficit hyperactivity disorder symptomatology (hyperactivity) and developmental motor coordination disorder traits should be appreciable in the first year of life, there do not yet exist reliable methods for ascertaining them in clinical settings, and these data underscore the importance of future research to establish such assessment methodologies in order to enable comprehensive early developmental profiles for the ascertainment of early autism risk to be specified in individual children. The inherited liabilities elucidated by these data represent not only indices of inherited susceptibility to autism, but parsimonious intervention targets for polygenic forms of the condition, as well as candidate trans diagnostic endophenotypes of potential relevance to a diversity of neuropsychiatric syndromes.

## Supplementary information

Supplemental Table 1 and Table 2
