# Supplementary material for: Behavioral predictors of autism recurrence are genetically independent and influence social reciprocity: evidence that polygenic ASD risk is mediated by separable elements of developmental liability
Source: Transl Psychiatry. 2019 Aug 22;9:202. doi: 10.1038/s41398-019-0545-z (PMC6706410; doi:10.1038/s41398-019-0545-z)
Supplement: Supplementary file 1 — Supplemental Table 1 and Table 2 [file 41398_2019_545_MOESM1_ESM.docx]

**Supplementary Materials**

| **Supplementary Table 1***. Selected Sample Characteristics of the California Twin Sample. Information on the Missouri sample has been previously reported ^1, 6^* | | | |
| --- | --- | --- | --- |
| Variable | Nonsample  (n=2,873) | Sample  (n=146) | Statistic |
| Age of Mother | 29.34(6.267) | 30.95(6.051) | t(2870)=3.013, p<0.003 |
| Age of Father | 31.65(7.215) | 33.59(7.328) | t(2628)=3.112, p<0.002 |
| Mother’s Education | 3.61(1.673) | 4.90(1.511) | t(2811)=9.138, p<0.000 |
| Father’s Education | 3.42(1.685) | 4.55(1.658) | t(2551)=7.790, p<0.000 |
| Number of Prenatal Care Visits | 13.16 (5.342) | 14.42 (4.988) | t(2793) = 2.732, p<0.006 |
| Father’s Race |  |  |  |
| Multiracial | 4.0% | 8.2% | X^2^ (19.703, 7), p<0.006 |
| Other | 3.9% | 0.7% |  |
| Unknown | 8.3% | 2.1% |  |
| How Family Paid for Prenatal Care |  |  |  |
| Private Insurance | 41.0% | 67.1% | X^2^ (47.195, 2), p<0.000 |
| Government Program | 57.5% | 28.8% |  |
| Self-Pay | 1.8% | 4.1% |  |
| Government Assistance | 66.8% | 40.0% | X^2^ (43.755,1), p<0.000 |
| Foreign Born Mother | 40.9% | 12.3% | X^2^ (47.152, 1), p<0.000 |

*Note.* The “Nonsample” data are from all Hispanic families living with twins in the California site born between January 1, 2012 and December 31, 2012. The “Sample” data are from the families who matriculated through both Wave 1 and Wave 2 of data collection at the California site. Proportions for significant chi-squared tests and means with standard deviations in parentheses are presented for significant t-tests. For t-tests, equal variances assumed statistics are reported except when denoted by asterisk. In these cases, the null hypothesis for Levene’s test for equality of variances was accepted; and therefore, the ‘equal variances not assumed’ statistic was reported.

**Supplementary Table 2**. Results of linear regression analysis examining the joint contribution of three behavioral predictors of autism recurrence (measured at 36-48 months) to variation in autism-related variation in early childhood reciprocal social behavior at 48 months.  Adjusted R square is reported for the full regression model, along with changes in adjusted R square that occur when a given individual behavioral trait is excluded from the model, and the result compared with that for the full model.

| Outcome Modeled | AdjR^2^ | BPAR | B | t | Sig | Δ Adj R^2^ |
| --- | --- | --- | --- | --- | --- | --- |
| SRS at 48 months | 0.30 | Biparental QAT | 0.155 | 2.262 | <0.025 | 0.02 |
|  |  | Variation in Attentional Impairment | 0.386 | 5.576 | <0.001 | 0.14 |
|  |  | Variation in Motor Coordination | -0.221 | -3.203 | <0.002 | 0.04 |
|  |  | Site | .079 | 1.173 | 0.243 | 0.00 |

SRS: Social Responsiveness Scale
